# Identifying critical differentiation state of MCF-7 cells for breast cancer by dynamical network biomarkers

**DOI:** 10.3389/fgene.2015.00252

**Published:** 2015-07-28

**Authors:** Pei Chen, Rui Liu, Luonan Chen, Kazuyuki Aihara

**Affiliations:** ^1^School of Computer Science, South China University of TechnologyGuangzhou, China; ^2^School of Mathematics, South China University of TechnologyGuangzhou, China; ^3^Collaborative Research Center for Innovative Mathematical Modelling, University of TokyoTokyo, Japan; ^4^Key Laboratory of Systems Biology, Innovation Center for Cell Signaling Network, Institute of Biochemistry and Cell Biology, Shanghai Institutes for Biological Sciences, Chinese Academy of SciencesShanghai, China

**Keywords:** cell differentiation, dynamical network biomarker (DNB), pre-transition state, critical transition, early-warning signal, breast cancer

## Abstract

Identifying the pre-transition state just before a critical transition during a complex biological process is a challenging task, because the state of the system may show neither apparent changes nor clear phenomena before this critical transition during the biological process. By exploring rich correlation information provided by high-throughput data, the dynamical network biomarker (DNB) can identify the pre-transition state. In this work, we apply DNB to detect an early-warning signal of breast cancer on the basis of gene expression data of MCF-7 cell differentiation. We find a number of the related modules and pathways in the samples, which can be used not only as the biomarkers of cancer cells but also as the drug targets. Both functional and pathway enrichment analyses validate the results.

## Introduction

Breast cancer, one of the most common cancers, is clearly a heterogeneous, complex, interrelated disease involving multi-factorial etiologies. The tumorgenesis of breast cancer is typically characterized by a combination of the interactions between environmental (external) factors and a genetically susceptible host (Ou et al., 2010). The prevalence of breast cancer as well as the growing economic and societal burden of the treatment is making it urgently necessary to implement interventions to prevent or at least delay the occurrence of breast cancer. However, it is still a challenging task to detect breast cancer in its early stage since it is usually silent and without clear symptoms in its initial stages, while irreversible complications may develop rapidly before the implementation of effective treatment (Saini et al., 2011). Many studies of breast cancer are based on MCF-7 cells. MCF-7 is the acronym of Michigan Cancer Foundation-7. The MCF-7 cells are cancer cells that are classified as invasive breast ductal carcinoma. Although the underlying molecular mechanism of the progression for MCF-7 cells is far from clear, it has been found that heregulin (HRG) and epidermal growth factor (EGF) are involved in inducing the critical transition of cell differentiation or proliferation (Normanno et al., 1994; Suzuki et al., 2004; Nagashima et al., 2007; Saeki et al., 2009). In this work, we quantitatively analyze time-course microarray data of MCF-7 cells, and identify the key genes, i.e., dynamical network biomarker (DNB), which may indicate the imminent critical transition of the cancer cells during cell differentiation or proliferation.

**Figure 1:**
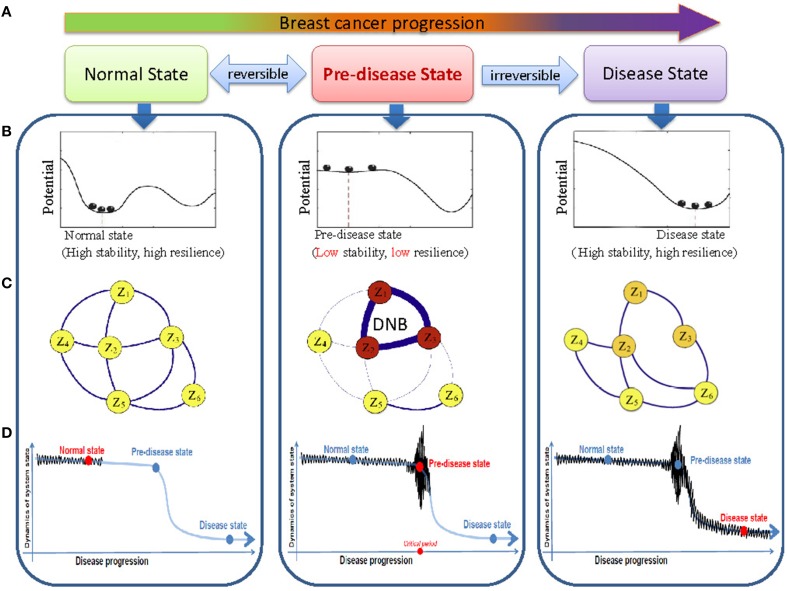
**The outline for identifying the transition state by DNB based on time-course data. (A)** The progression of breast cancer cells can be divided into three states, i.e., the before-transition state, the pre-transition state, and the after-transition state. **(B)** A system at the before-transition state or the after-transition state is stable with high resilience, while it is unstable with low resilience when it is at the pre-transition state. **(C)** In the pre-transition state, large fluctuations of the DNB members are correlated strongly. This critical phenomenon do not appear at the before-transition and the after-transition states. **(D)** The DNB members show large fluctuations in their expressions at the pre-transition state, compared with smaller fluctuations of the expressions at the before-transition and the after-transition states.

We previously hypothesized that a complex biological process (e.g., disease progression) can be divided into three stages or states (Figures 1A,B): (A) a before-transition stage (or a normal state) with high resilience and robustness to perturbations; (B) a pre-transition stage (or a pre-disease state), just before the critical transition to the disease state, i.e., occurring before an imminent phase transition point is reached, therefore, with low resilience and robustness due to its dynamical structure; (C) an after-transition stage (or a disease state), representing a seriously deteriorated stage possibly with high resilience and robustness again, because it is generally difficult for the system at this state to recover or return to the normal state even after treatment (Chen et al., 2012; Liu et al., 2012a). This classification is supported by the observations that there exist catastrophic shifts during the progression of many chronic diseases, i.e., the sudden deterioration of diseases (Litt et al., 2001; McSharry et al., 2003; Venegas et al., 2005; Hirata et al., 2010; He et al., 2012). A drastic or qualitative transition in a focal system or network, from a normal state to a disease state, corresponds to a so-called bifurcation point in dynamical systems theory (Gilmore, 1993; Murray, 2002). When the system is near a bifurcation point, or a critical point, there exists a dominant group, which we called as the DNB. The DNB can be defined by the following three conditions (Chen et al., 2012): The correlation between any pair of members in DNB becomes very strong; The correlation between one member of DNB and any other molecule of non-DNB becomes very weak; Any member of DNB becomes highly fluctuating. The DNB is not only a theoretical concept, but also has been successfully applied to real biological data, and used to identified the early-warning signals of sudden deterioration of several complex diseases (Li et al., 2013; Liu et al., 2013a,b, 2014a; Zeng et al., 2014; Tan et al., 2015).

In this work, by applying the DNB approach to the datasets of MCF-7 breast cancer cell line (GSE13009, GSE6462, and GSE10145), we identify the DNB members composed by a group of genes that may indicate the imminent critical transition during the progression of breast cancer cells.

## Methods

We first describe the theoretical basis, i.e., the DNB theory, and then provide the procedures used to preprocess input datasets and implement the detail DNB score algorithm.

### Theoretical basis

As explained in Section Introduction, a biological process can be generally divided into the three stages, i.e., (A) the before-transition state (or normal state in complex diseases), (B) the pre-transition state (or pre-disease state in complex diseases) and (C) the after-transition state (or disease state in complex diseases) (Figure 1A). The before-transition state is a stable state representing a stable stage with high resilience, during which the state may change gradually. The pre-transition state is a state defined as the limit of the before-transition state just before a critical transition. This state is considered to be still reversible to the before-transition state since appropriate external interventions can drive it back to the before-transition state relatively easily. However, further progression beyond the pre-transition state will result in a drastic transition to the after-transition state, another stable state, and it is difficult to return to the before-transition state even with intensive interventions. The after-transition state represents a seriously ill stage in complex diseases.

Different from the traditional biomarkers, e.g., molecular biomarkers and network biomarkers (Liu et al., 2012b; Wen et al., 2014; Zhang et al., 2014, 2015), which are designed to distinguish the disease samples from normal samples and thus reflect the severity or presence of the illness at the disease state, the DNB theory aims to distinguish the pre-disease samples from normal samples according to the critical dynamical behavior of DNB molecules (Liu et al., 2014b). In other words, the DNB method is designed to identify a group of strongly correlated and significantly fluctuating molecules, which are also called “the leading network” because those genes may lead the transition of the whole system from the normal state to the disease state (Liu et al., 2012a).

Although elucidating the critical transition at the network level holds the key to understand the fundamental mechanism of disease development or cell differentiation, it is notably hard to reliably identify the pre-transition state because there may be little apparent difference between the before-transition and pre-transition states. This is also the reason why diagnosis based on traditional biomarkers may fail to indicate the pre-transition state. The theoretical basis for detecting DNB is summarized by the following conditions (Figures 1C,D), which have been proven to hold simultaneously when the system approaches the pre-transition state (Chen et al., 2012):

1. Deviations of a group of molecules called DNB among the whole population of molecules, drastically increases (the fluctuation condition);
2. Correlation between any two molecules among DNB increases (the internal correlation condition);
3. Correlation between any molecule in DNB and another in non-DNB decreases (the external correlation condition);
4. There are no drastic changes for deviations and correlations of molecules among the remaining molecules of the system, i.e., non-DNB.

Dynamics satisfying the preceding conditions can be viewed as locally herding behavior, i.e., members in a DNB subnetwork act together with strongly correlated fluctuation. These conditions imply an imminent regime shift or a phase transition, and therefore, can be used to signal the impending emergence of the critical transition. Such a phenomenon can also be described as the DNB molecules get dynamically correlated or connected so that the system can be reorganized in a different way.

### Data processing and algorithm

Three gene expression profiling datasets were downloaded from the NCBI GEO database (ID: GSE13009, GSE6462, and GSE10145) (www.ncbi.nlm.nih.gov/geo). In these datasets, probe sets without corresponding gene symbols were not considered in our analysis. The expression values of probe sets mapped to the same gene were averaged. Genes in the DNBs for the three datasets were linked and correlated by the combined functional couplings among them from various databases of protein-protein interactions of STRING, FunCoup, and BioGrid. In each disease dataset, the expression profiling information was mapped to the integrated networks individually for identifying the corresponding DNB. For each species, we downloaded the biomolecular interaction networks from various databases, including BioGrid (http://www.thebiogrid.org), TRED, KEGG (http://www.genome.jp/kegg), and HPRD (http://www.hprd.org). First, the available functional linkage information for Mus musculus and Homo sapiens was downloaded from these databases and combined. For instance, after removing any redundancy in dataset GSE13009, we obtained 37,950 linkages in 13785 human genes. Next, the genes evaluated in these microarray datasets were mapped individually to their integrated functional linkage networks. In order to trigger critical changes, MCF-7 cells were exposed to growth factors heregulin (HRG) for up to 6 h and the temporal expression of transcription factors was monitored (Saeki et al., 2009). There were the case group and the control group for the experiment. For the case group, the gene expressions were recorded respectively in 17 time points (10 min, 15 min, 20 min, 30 min, 45 min, 1 h, 1 h 30 min, 2 h, 3 h, 4 h, 6 h, 8 h, 12 h, 24 h, 36 h, 48 h, and 72 h). The networks were visualized using Cytoscape (www.cytoscape.org) and a part of the functional analysis was based on Integrate and understand complex omics data (IPA). The detailed algorithm is given in the Supplementary Materials.

## Results

### The identified DNB and the pre-transition state

**Figure 2:**
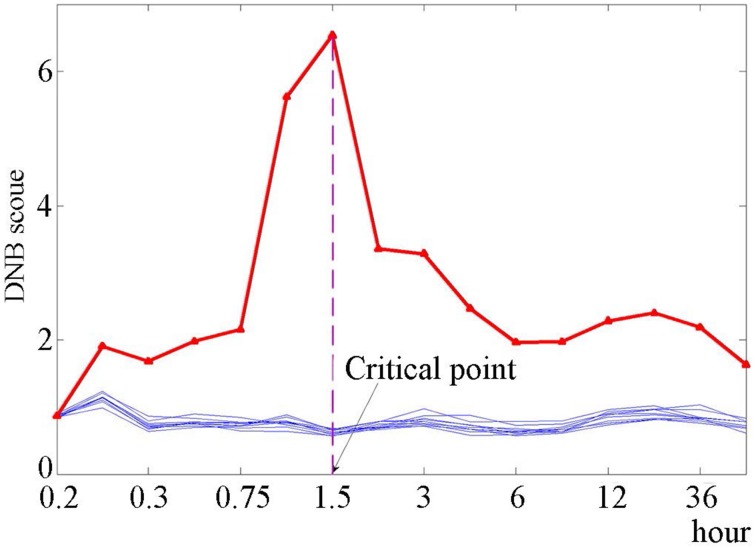
**The DNB scores for the identified dominant group and bootstrap groups**. The DNB scores are shown for the identified dominant group (red curve) and 10 groups from bootstrap (blue curves). It can be seen that for the red curve, the DNB score increases sharply from the 6th point (1 h) and reaches the peak at the 7th point (1.5 h). For the bootstrap analysis, we randomly selected 10 gene sets, each of which is composed of the same number of members as the dominant group. Then the DNB score was calculated for each randomly chosen group.

Applying the DNB method to dataset GSE13009, the DNB containing 104 genes was identified for HRG-induced differentiation of cancer cells. We listed all of the identified DNB members in Supplementary Table S1 “Detail description of the identified DNB.” The process of identifying the DNB can be found in “The algorithm for identifying the DNB” of Supplementary Materials. During the progression of cancer cells, we also identified the pre-transition state between the before-transition state and the after-transition state (Figure 2), which is the critical stage when the progression of MCF-7 cells is just before the differentiation triggered by HRG (Nagashima et al., 2007). Actually, based on Figure 2, the sharp increase of the DNB score (the red curve) represents an indicative early-warning signal 1–1.5 h after the expose to HRG, and thus before the differentiation detected by molecular markers. In fact, the original assay showed that the AP-1 complex in HRG-treated MCF-7 cells contains c-JUN, c-FOS, and FRA-1, although the association of c-JUN in the complex is transient (Saeki et al., 2009). Besides, the stimulation of MCF-7 breast cancer cells with EGF and HRG resulted in very similar early transcription profiles up to 90 min; however, subsequent cellular phenotypes differed after 3 h (Saeki et al., 2009), which suggests that the differentiation is around 3 h (the 9th sampling time point). The bootstrap validation (the blue curves) is also known in Figure 2, which exhibits that the randomly chosen groups containing the same number of genes with DNB are insensitive to the critical transition.

**Figure 3:**
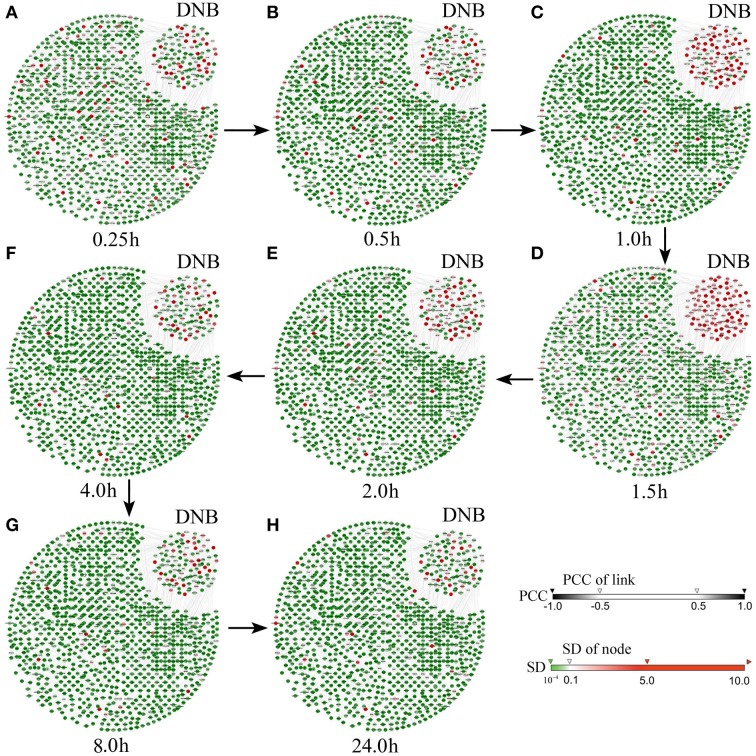
**Dynamical changes in the network including the selected DNB during the progression of HRG-induced breast cancer**. The figures show the dynamical changes of the molecular network at **(A)** 0.25 h, **(B)** 0.5 h, **(C)** 1 h, **(D)** 1.5 h, **(E)** 2 h, **(F)** 4 h, **(G)** 8 h, and **(H)** 24 h. It can be seen that, the DNB members are correlated strongly while each member shows large fluctuation in its expression during 1–1.5 h. This critical phenomenon does not appear before or after this period, i.e., the before-transition or the after-transition state. Thus, the pre-transition stage is around 1–1.5 h, just before the cell differentiation triggered by HRG (7).

Figure 3 shows the dynamical evolution in the whole feature network based on the case data. It can be seen from Figure 3 that the selected 104 genes (the top right corner in each network) are strongly correlated with large fluctuations 1–1.5 h before the critical transition, which provides a significant signal indicating the pre-transition stage of cell differentiation, while other genes show no significant signal. Clearly, when the differentiation is impending, these selected genes form a special subnetwork, the so-called DNB, which makes the first move from the before-transition state toward the after-transition state during the transition. Interestingly, members of the DNB behaved similarly to other genes after the system moved to the after-transition state. It can be seen that, on the other hand, neither the whole gene network nor the DNB presents a signal before or after the transition, which shows the sensitivity of the DNB method only at the pre-transition state. In fact, the DNB method reveals the existence of the pre-transition state, which, however, may not be detected by molecules such as EGR4, FOSL-1, FHL2, and DIPA, although these four transcription factors are proved to be effective for indicating the differentiation of breast cancer cells (Saeki et al., 2009). In other words, the molecular biomarkers cannot provide early-warning signals before the cell differentiation (at 3 h, or the 9th sampling time point). Therefore, the benefits brought by the DNB method in signaling the pre-transition state make the identification and management of high-risk cases effective.

### Validation

**Figure 4:**
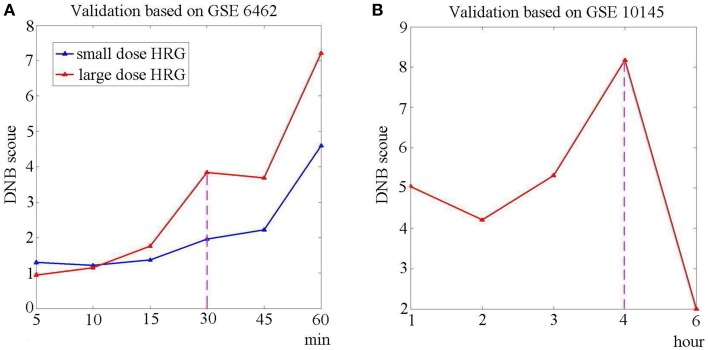
**The validation of DNB based on independent datasets**. To validate the sensitivity and effectiveness, we calculated the DNB score using the identified genes, based on two independent dataset. **(A)** The DNB scores based on GSE6462. The red curve represents the case of large dose HRG usage (1and 10 nM), while the blue curve stands for the case of small dose HRG expose (0.1 and 0.5 nM). It can be seen that there is a signal at the 4th sampling point (30 min) when the MCF-7 cells are exposed to large dose of HRG. **(B)** The DNB score based on GSE10145. The curve shows that a peak of DNB score is at the 4th sampling point (4 h).

Hereto we have shown the sensitivity and effectiveness of the identified DNB. Figures 4A,B respectively show the DNB scores based on independent datasets GSE6462 and GSE10145. From dataset GSE6462, it can be seen from Figure 4A that the identified DNB also showed a signal for large dose (1 and 10 nM) HRG expose at the 4th sampling point (30 min), while there is no clear signal for small dose (0.1 and 0.5 nM) HRG expose. It agrees with the original experiments (Nagashima et al., 2007) that HRG-induced cellular differentiation of MCF-7 cells is observed around 60 min. From Figure 4B, it can be seen that the signal is detected by the DNB score at the 4th time point, which also agrees with the observations and shows the sensitivity of the identified DNB. The bootstrap analysis for both datasets is shown in Figure S1 of Supplementary Materials.

### Functional analysis

Heregulin (HRG) can induce dose-dependent transient and sustained intracellular signaling, proliferation, and differentiation of MCF-7 breast cancer cells (Barlund et al., 2002; Huang et al., 2009). In the infected host, some metabolic pathways responded to these interruptions and became increasingly disordered. The following results show that some reported phenomena were consistent with our investigations, which also provides novel insights into the biological processes.

The identified DNB module is related to the regulation of an apoptotic process (GO:0042981) with the significant *P*-value (2.93E-06), the regulation of the programmed cell death (GO:0043067) with the significant *P*-value (4.10E-05) and the regulation of the cell death (GO:0010941) with the significant *P*-value (7.41E-04) by the website tool DAVID Bioinformatics Resource (Huang et al., 2009). By the pathway analysis in the KEGG database, we found that seven genes (CEBPA, SMAD3, GSK3B, LAMC2, MMP1, PIK3R3, and RXRA) in this DNB module participate in cancer pathways, and many genes of this module also take part in other cancer-related pathways, e.g., the Wnt signaling pathway with *P*-value (9.10E-03), the p53 signaling pathway with *P*-value (1.10E-04), and the ECM-receptor interaction with *P*-value (2.30E-03).

Many genes in this DNB module have been proved to be related to a cancer or tumor process, and in particular, some of these genes are associated with breast cancer. For example, BCAS4 is an important gene for breast tumor development and progression (Barlund et al., 2002). ARID3B is one of genes which regulates cell motility and actin cytoskeleton organization (Casanova et al., 2011) and is found to be associated with breast cancer onset (Akhavantabasi et al., 2012). TNFRSF21 encodes a tumor necrosis factor receptor, which can regulate the NF-kappaB and mediate an apoptosis process (Kasof et al., 2001). LAMC2 encodes the gamma chain isoform laminin, which is involved in many biological processes, and LAMC2 is also proved to be related to the breast cancer process (Sathyanarayana et al., 2003; Koshikawa et al., 2005). Therefore, DNB for HRG-induced breast cancer can mainly induce cancer by affecting the processes of regulation of apoptosis, regulation of programmed cell death and regulation of cell death.

## Discussion

Breast cancer is a progressive disease and its deterioration course is primarily characterized by cancer cell differentiation or proliferation, which significantly damages the health of women all over the world. Detecting the early-warning signal of the cell differentiation of cancer cells provides an opportunity to interrupt and prevent the continuing costly cycle of managing breast cancer and its complications. The critical transition of cancer cells involving proliferation or differentiation can be induced by a ligand of the ErbB family receptor, heregulin, which evokes kinase activity of MCF-7 cells. Actually, in MCF-7, HRG induced graded signaling and early transcription, followed by auto-induction of multiple positive/negative feedback mechanisms, and prolongation of signaling activity might switch cells irreversibly (Saeki et al., 2009). It is an important future problem to analyses whether the HRG-induced critical transition is reversible in the pre-transition state.

In this work, we applied the DNB method to the identification of the pre-transition state on the basis of a composition of microarray data from the breast cancer cell line. First, we introduced the DNB approach which aims at detecting the critical signals of the cell differentiation and indicating the pre-transition state or stage. Second, based on the cell line data, we identified the pre-transition stage right before the cell differentiation induced by heregulin (HRG) during the progression of cancer cells. Actually, an indicative early-warning signal is presented by DNB at 1 h after the expose to HRG. The validation based on bootstrap (Figure 2) and other two datasets (Figure 4) demonstrated the sensitivity and effectiveness of the identified DNB for the HRG triggered differentiation. Besides, we showed that some metabolic pathways responded to the HRG-induced interruptions and became increasingly disordered during the biological process. Therefore, the DNB method provides a new way to pry into the underlying mechanism of cell differentiation and thus is helpful to achieve the timely intervention. This is the main value in the potential applications of the DNB method from a network point of view.

On the other hand, there are limitations of this work. First, the validity of the identified pre-transition state and the DNB needs further supports from biological experiments and clinical studies. Second, the method is insensitive when the genes are not differentially expressed (see the algorithm stated in the Supplementary Material). The algorithm is also needed to be further improved on the aspects of both sensitivity and accuracy. Although this work is merely a step toward detecting the early-warning signals of critical transition during cancer cell progression of breast cancer and the algorithm is expected to be improved in both time saving and capacity efficient ways, it opens a window of an opportunity for experimental and clinical study on the early-warning system of breast cancer.

### Conflict of interest statement

The authors declare that the research was conducted in the absence of any commercial or financial relationships that could be construed as a potential conflict of interest.
