# Supplementary material for: Identifying critical differentiation state of MCF-7 cells for breast cancer by dynamical network biomarkers
Source: Front Genet. 2015 Jul 28;6:252. doi: 10.3389/fgene.2015.00252 (PMC4516973; doi:10.3389/fgene.2015.00252)
Supplement: Supplementary file 1 [file DataSheet1.DOCX]

## Supplementary Materials

Supplementary Material 1: The algorithm for identifying the DNB

Step 1: Choose differential expression genes from the high-throughput data. Here, the expression means the gene expression microarray data for each gene. At each sampling point, we carry out the student t-test with the significance level p<0.05, and the false discovery rate (FDR) which is a statistical method used in multiple hypothesis testing to correct for multiple comparisons (e.g., student t-test) and measured by $FDR = E \left[ \frac{V}{R} \right]$, where V is the number of false positives (Type I error), R is the number of rejected null hypotheses. Then we screen out differential expression molecules, i.e., the concentration values of the selected features in a case group are significantly different (in the sense of mean values) from those in control group. In Supplementary Table 1, we have given the p-value for each identified gene through the student t-test and FDR.

Step 2: Based on each set of the selected differential expression molecules, employing two-fold change screening, we obtain genes respectively for each sampling time points, which exhibit relatively significant changes in the standard deviation in each period

Step 3: Cluster variables at each sampling time point by correlations. Among all clusters, determine the dominant group or the DNB by significance analysis, where the DNB score is calculated with the normalized values as follows:

$$I=\frac{SD\cdot PCC_{in}}{PCC_{out}+\epsilon} ,$$

where $SD$ is the average standard deviation of all variables in DNB; $PCC_{in}$ is the average Pearson's correlation coefficient between variables in DNB in absolute values; $PCC_{out}$ is the average Pearson's correlation coefficient between a variable inside DNB and another one outside in absolute values; and $\epsilon$ is a small positive constant to avoid zero division.

Supplementary Material 2:


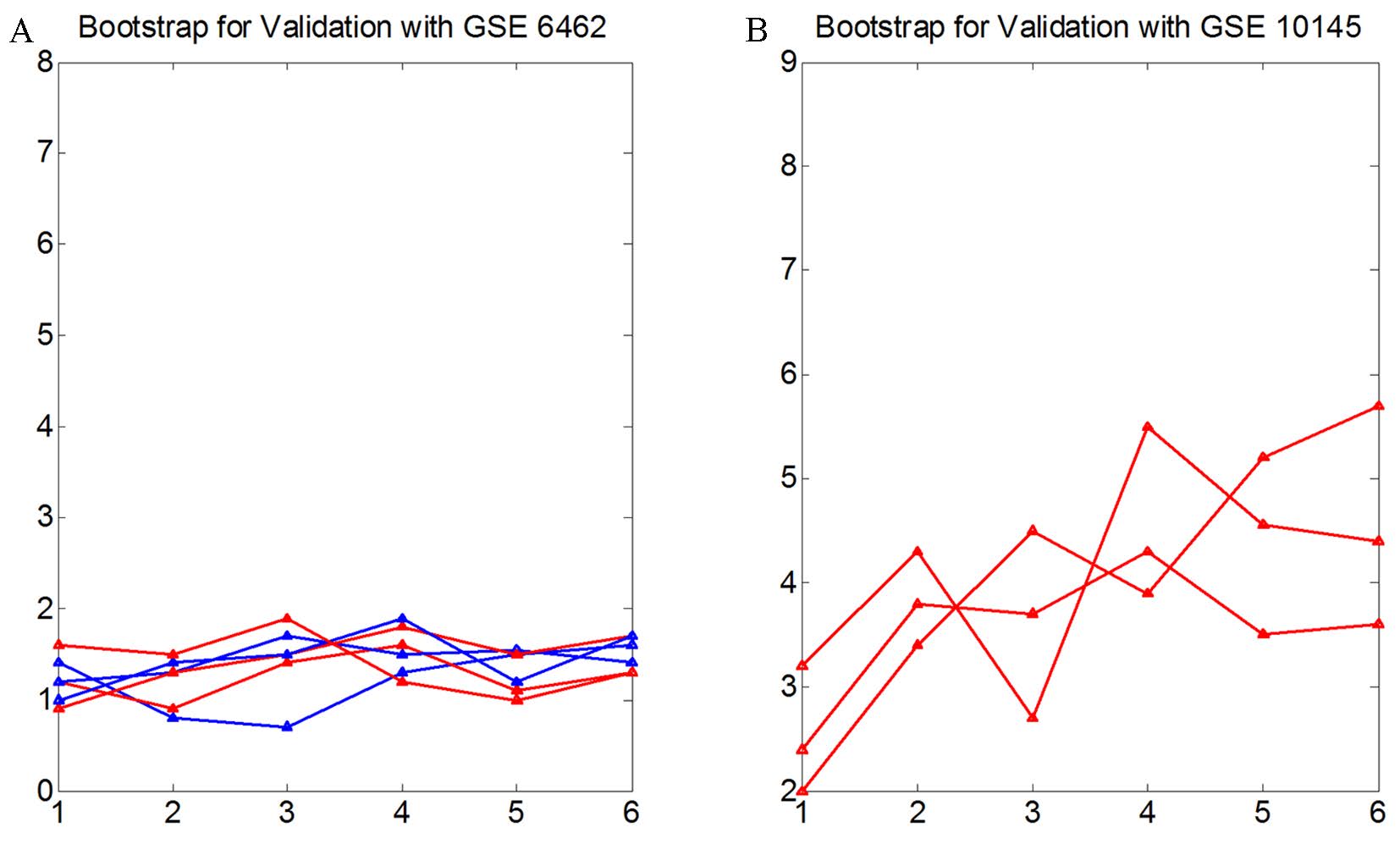


Figure S1: The bootstrap analysis for the validation datasets.

To validate the sensitivity of identified genes, we carried out the bootstrap analysis for the two validation datasets, i.e., we randomly selected several group of gene sets, each of which is composed of the same number of members as the DNB group. Then the DNB score is calculated for each randomly chosen group. (a) The bootstrap based on GSE6462. The red curves represent the case of large dose HRG usage (1nM and 10nM), while the blue curves stand for the case of small dose HRG expose (0.1nM and 0.5nM). (b) The bootstrap based on GSE10145. From both bootstrap results, it can be seen that the randomly chosen genes are insensitive to the DNB approach.

Supplementary Material 3:

Table S1 Detail description of the identified DNB

| Gene | Description | Location | Family | Drugs | P-value |
| --- | --- | --- | --- | --- | --- |
| DNAJB1 | DnaJ (Hsp40) homolog, subfamily B, member 1 | Nucleus | other |  | 0.000088 |
| EPHA2 | EPH receptor A2 | Plasma Membrane | kinase | dasatinib, regorafenib, dasatinib/dexamethasone/vincristine | 0.00030 |
| CLCF1 | cardiotrophin-like cytokine factor 1 | Extracellular Space | cytokine |  | 0.00032 |
| AMD1 | adenosylmethionine decarboxylase 1 | Cytoplasm | enzyme |  | 0.00019 |
| PHLDA1 | pleckstrin homology-like domain, family A, member 1 | Cytoplasm | other |  | 0.00095 |
| CCNG2 | cyclin G2 | Nucleus | other |  | 0.00013 |
| NOP16 | NOP16 nucleolar protein | Nucleus | other |  | 0.00097 |
| LDLR | low density lipoprotein receptor | Plasma Membrane | transporter |  | 0.00096 |
| PIP5K1A | phosphatidylinositol-4-phosphate 5-kinase, type I, alpha | Cytoplasm | kinase |  | 0.00030 |
| PSME4 | proteasome (prosome, macropain) activator subunit 4 | Cytoplasm | other |  | 0.0011 |
| GPR87 | G protein-coupled receptor 87 | Plasma Membrane | G-protein coupled receptor |  | 0.0023 |
| ARID3B | AT rich interactive domain 3B (BRIGHT-like) | Nucleus | other |  | 0.00061 |
| F2RL1 | coagulation factor II (thrombin) receptor-like 1 | Plasma Membrane | G-protein coupled receptor |  | 0.0028 |
| BCAS4 | breast carcinoma amplified sequence 4 | Cytoplasm | other |  | 0.0081 |
| STX3 | syntaxin 3 | Plasma Membrane | transporter |  | 0.00073 |
| YKT6 | YKT6 v-SNARE homolog (S. cerevisiae) | Cytoplasm | enzyme |  | 0.0015 |
| SOWAHC | sosondowah ankyrin repeat domain family member C | Nucleus | transcription regulator |  | 0.00076 |
| ARL14 | ADP-ribosylation factor-like 14 | Other | other |  | 0.0027 |
| HBEGF | heparin-binding EGF-like growth factor | Extracellular Space | growth factor |  | 0.0032 |
| SMTN | smoothelin | Extracellular Space | other |  | 0.0011 |
| GDF15 | growth differentiation factor 15 | Extracellular Space | growth factor |  | 0.0062 |
| TBC1D31 | TBC1 domain family, member 31 | Cytoplasm | other |  | 0.017 |
| CLDN4 | claudin 4 | Plasma Membrane | transmembrane receptor |  | 0.008 |
| SERPINH1 | serpin peptidase inhibitor, clade H (heat shock protein 47), member 1, (collagen binding protein 1) | Extracellular Space | other |  | 0.042 |
| KLF6 | Kruppel-like factor 6 | Nucleus | transcription regulator |  | 0.023 |
| VCL | vinculin | Plasma Membrane | enzyme |  | 0.019 |
| FHL2 | four and a half LIM domains 2 | Nucleus | transcription regulator |  | 0.036 |
| IFRD1 | interferon-related developmental regulator 1 | Nucleus | other |  | 0.0061 |
| ATF3 | activating transcription factor 3 | Nucleus | transcription regulator |  | 0.0034 |
| SMAD6 | SMAD family member 6 | Nucleus | transcription regulator |  | 0.0045 |
| GAL | galanin/GMAP prepropeptide | Extracellular Space | other |  | 0.017 |
| CLN8 | ceroid-lipofuscinosis, neuronal 8 (epilepsy, progressive with mental retardation) | Cytoplasm | other |  | 0.0059 |
| REEP5 | receptor accessory protein 5 | Extracellular Space | transporter |  | 0.013 |
| RND3 | Rho family GTPase 3 | Cytoplasm | enzyme |  | 0.016 |
| KIF18B | kinesin family member 18B | Cytoplasm | other |  | 0.048 |
| NDUFAF4 | NADH dehydrogenase (ubiquinone) complex I, assembly factor 4 | Cytoplasm | other |  |  |
| MAK16 | MAK16 homolog (S. cerevisiae) | Nucleus | other |  | 0.050 |
| PSME3 | proteasome (prosome, macropain) activator subunit 3 (PA28 gamma; Ki) | Cytoplasm | peptidase |  | 0.0039 |
| C3orf52 | chromosome 3 open reading frame 52 | Other | other |  | 0.0057 |
| STK17A | serine/threonine kinase 17a | Nucleus | kinase |  | 0.0013 |
| GEM | GTP binding protein overexpressed in skeletal muscle | Plasma Membrane | enzyme |  | 0.010 |
| ARHGAP12 | Rho GTPase activating protein 12 | Cytoplasm | other |  | 0.0064 |
| TOMM34 | translocase of outer mitochondrial membrane 34 | Cytoplasm | other |  | 0.0038 |
| TRIB1 | tribbles pseudokinase 1 | Cytoplasm | kinase |  | 0.0065 |
| TNFRSF21 | tumor necrosis factor receptor superfamily, member 21 | Plasma Membrane | transmembrane receptor |  | 0.012 |
| NPC1 | Niemann-Pick disease, type C1 | Cytoplasm | transporter |  | 0.0093 |
| FLNB | filamin B, beta | Cytoplasm | other |  | 0.012 |
| LAMC2 | laminin, gamma 2 | Extracellular Space | other |  | 0.0076 |
| ISG20 | interferon stimulated exonuclease gene 20kDa | Nucleus | enzyme |  | 0.012 |
| BZW1 | basic leucine zipper and W2 domains 1 | Cytoplasm | translation regulator |  | 0.0070 |
| CDC42EP3 | CDC42 effector protein (Rho GTPase binding) 3 | Cytoplasm | other |  | 0.011 |
| MAP7D1 | MAP7 domain containing 1 | Cytoplasm | other |  | 0.0064 |
| INPP1 | inositol polyphosphate-1-phosphatase | Cytoplasm | phosphatase |  | 0.0061 |
| HMGCR | 3-hydroxy-3-methylglutaryl-CoA reductase | Cytoplasm | enzyme | aspirin/pravastatin, ezetimibe/fluvastatin, atorvastatin/niacin, atorvastatin/ezetimibe, ezetimibe/rosuvastatin, simvastatin/sitagliptin, choline fenofibrate/simvastatin, fenofibrate/simvastatin, pitavastatin, lovastatin/niacin, ezetimibe/simvastatin, amlodipine/atorvastatin, fluvastatin, cerivastatin, atorvastatin, pravastatin, simvastatin, lovastatin, mevastatin, rosuvastatin | 0.013 |
| ARHGAP29 | Rho GTPase activating protein 29 | Cytoplasm | other |  | 0.0037 |
| NAB2 | NGFI-A binding protein 2 (EGR1 binding protein 2) | Nucleus | transcription regulator |  | 0.014 |
| NR4A3 | nuclear receptor subfamily 4, group A, member 3 | Nucleus | ligand-dependent nuclear receptor |  | 0.017 |
| GSK3B | glycogen synthase kinase 3 beta | Nucleus | kinase | enzastaurin | 0.026 |
| CEBPA | CCAAT/enhancer binding protein (C/EBP), alpha | Nucleus | transcription regulator |  | 0.013 |
| CTSV | cathepsin V | Cytoplasm | peptidase |  |  |
| LRP8 | low density lipoprotein receptor-related protein 8, apolipoprotein e receptor | Plasma Membrane | transmembrane receptor |  | 0.014 |
| CLDN7 | claudin 7 | Plasma Membrane | other |  | 0.011 |
| PMAIP1 | phorbol-12-myristate-13-acetate-induced protein 1 | Cytoplasm | other |  | 0.019 |
| PIK3R3 | phosphoinositide-3-kinase, regulatory subunit 3 (gamma) | Cytoplasm | kinase |  | 0.0092 |
| SMAD3 | SMAD family member 3 | Nucleus | transcription regulator |  | 0.017 |
| PICALM | phosphatidylinositol binding clathrin assembly protein | Cytoplasm | other |  | 0.013 |
| PCLO | piccolo presynaptic cytomatrix protein | Cytoplasm | transporter |  | 0.023 |
| MC4R | melanocortin 4 receptor | Plasma Membrane | G-protein coupled receptor |  | 0.035 |
| KLF10 | Kruppel-like factor 10 | Nucleus | transcription regulator |  | 0.017 |
| RBMS1 | RNA binding motif, single stranded interacting protein 1 | Nucleus | other |  | 0.018 |
| TOMM22 | translocase of outer mitochondrial membrane 22 homolog (yeast) | Cytoplasm | transporter |  | 0.037 |
| SLC19A2 | solute carrier family 19 (thiamine transporter), member 2 | Plasma Membrane | transporter |  | 0.013 |
| CBLL1 | Cbl proto-oncogene-like 1, E3 ubiquitin protein ligase | Nucleus | enzyme |  | 0.018 |
| CUEDC1 | CUE domain containing 1 | Other | other |  | 0.0081 |
| CD44 | CD44 molecule (Indian blood group) | Plasma Membrane | enzyme |  | 0.012 |
| CSGALNACT2 | chondroitin sulfate N-acetylgalactosaminyltransferase 2 | Cytoplasm | enzyme |  | 0.0075 |
| FLRT3 | fibronectin leucine rich transmembrane protein 3 | Plasma Membrane | other |  | 0.021 |
| ADORA2B | adenosine A2b receptor | Plasma Membrane | G-protein coupled receptor | adenosine, enprofylline, dyphylline, aspirin/butalbital/caffeine, acetaminophen/caffeine/dihydrocodeine, acetaminophen/aspirin/caffeine, caffeine/ergotamine, aspirin/caffeine/propoxyphene, aspirin/butalbital/caffeine/codeine, aspirin/caffeine/dihydrocodeine, acetaminophen/butalbital/caffeine, aminophylline, aspirin/caffeine/orphenadrine, acetaminophen/butalbital/caffeine/codeine, theophylline, caffeine, acetaminophen/caffeine/chlorpheniramine/hydrocodone/phenylephrine | 0.024 |
| ATP6V0B | ATPase, H+ transporting, lysosomal 21kDa, V0 subunit b | Cytoplasm | transporter |  | 0.032 |
| PPP2R2A | protein phosphatase 2, regulatory subunit B, alpha | Cytoplasm | phosphatase |  | 0.039 |
| PNP | purine nucleoside phosphorylase | Nucleus | enzyme | forodesine, PD 141955 | 0.021 |
| PER2 | period circadian clock 2 | Nucleus | transcription regulator |  | 0.015 |
| YRDC | yrdC N(6)-threonylcarbamoyltransferase domain containing | Other | other |  | 0.017 |
| EIF5A | eukaryotic translation initiation factor 5A | Cytoplasm | translation regulator |  | 0.030 |
| RXRA | retinoid X receptor, alpha | Nucleus | ligand-dependent nuclear receptor | etretinate, daunorubicin/tretinoin, idarubicin/tretinoin, doxorubicin/tretinoin, bexarotene, acitretin, tretinoin, alitretinoin | 0.042 |
| RAB32 | RAB32, member RAS oncogene family | Cytoplasm | enzyme |  | 0.045 |
| SEC14L2 | SEC14-like 2 (S. cerevisiae) | Cytoplasm | transporter |  | 0.034 |
| FAIM | Fas apoptotic inhibitory molecule | Plasma Membrane | other |  | 0.042 |
| MMP1 | matrix metallopeptidase 1 (interstitial collagenase) | Extracellular Space | peptidase | marimastat | 0.035 |
| WEE1 | WEE1 G2 checkpoint kinase | Nucleus | kinase | MK 1775 | 0.029 |
| CCR2 | chemokine (C-C motif) receptor 2 | Plasma Membrane | G-protein coupled receptor |  | 0.024 |
| SERTAD3 | SERTA domain containing 3 | Nucleus | transcription regulator |  | 0.044 |
| PPP1R15A | protein phosphatase 1, regulatory subunit 15A | Cytoplasm | other |  | 0.028 |
| PLOD3 | procollagen-lysine, 2-oxoglutarate 5-dioxygenase 3 | Cytoplasm | enzyme |  | 0.039 |
| LPIN1 | lipin 1 | Nucleus | phosphatase |  | 0.031 |
| NIPBL | Nipped-B homolog (Drosophila) | Nucleus | transcription regulator |  | 0.020 |
| ACOT7 | acyl-CoA thioesterase 7 | Cytoplasm | enzyme |  | 0.032 |
| HYAL2 | hyaluronoglucosaminidase 2 | Cytoplasm | enzyme | hyaluronic acid, hylan | 0.026 |
| THBD | thrombomodulin | Plasma Membrane | transmembrane receptor |  | 0.025 |
| KRT86 | keratin 86, type II | Cytoplasm | other |  | 0.027 |
| SLC31A2 | solute carrier family 31 (copper transporter), member 2 | Plasma Membrane | transporter |  | 0.042 |
