# Supplementary figures and images for: Identifying critical differentiation state of MCF-7 cells for breast cancer by dynamical network biomarkers
Source: Front Genet. 2015 Jul 28;6:252. doi: 10.3389/fgene.2015.00252 (PMC4516973; doi:10.3389/fgene.2015.00252)

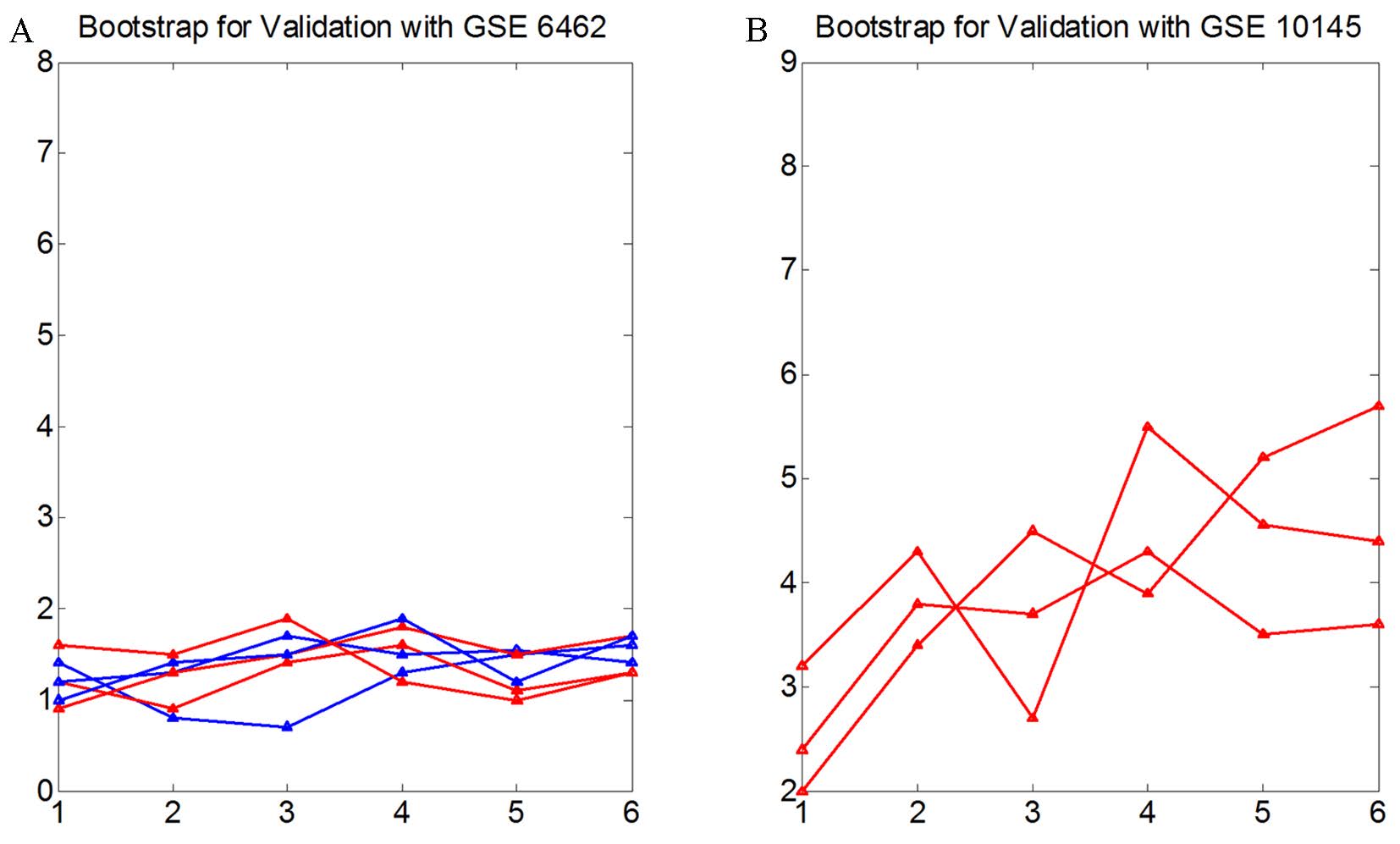

Supplement: Supplementary file 2 [file Image1.JPEG]
